# Pharmaceutical Care Network Europe definition of quality indicators for pharmaceutical care: a systematic literature review and international consensus development

**DOI:** 10.1007/s11096-023-01631-8

**Published:** 2023-08-30

**Authors:** Kenji Fujita, Kjell H. Halvorsen, Noriko Sato, Janja Jazbar, Pilar Modamio, Isabel Waltering, Isabelle De Wulf, Tommy Westerlund, Timothy F. Chen, Martina Teichert

**Affiliations:** 1 Kolling Institute, Faculty of Medicine and Health, The University of Sydney and the Northern Sydney Local Health District, Sydney, NSW Australia; 2 Department of Pharmacy, Faculty of Health Sciences, UiT The Arctic University of Norway, Tromsö, Norway; 3 School of Pharmacy, Faculty of Medicine and Health, The University of Sydney, Sydney, NSW Australia; 4 Faculty of Pharmacy, University of Ljubljana, Ljubljana, Slovenia; 5 Clinical Pharmacy and Pharmaceutical Care Unit, Department of Pharmacy and Pharmaceutical Technology, and Physical Chemistry, Faculty of Pharmacy and Food Sciences, University of Barcelona, Barcelona, Spain; 6 Institute for Pharmaceutical and Medicinal Chemistry, Clinical Pharmacy, University of Muenster, Münster, Germany; 7 Association of Belgian Pharmacists, Brussels, Belgium; 8 Department of Biomedical Science, Faculty of Health and Society, Malmö University, Malmö, Sweden; 9 Department of Public Health and Primary Care, Leiden University Medical Centre, Leiden, the Netherlands

**Keywords:** Consensus, Delphi technique, Pharmaceutical care, Quality indicators, Systematic review

## Abstract

**Background:**

Over the past 40 years, the tasks of pharmacists have shifted from logistic services to pharmaceutical care (PhC). Despite the increasing importance of measuring quality of care, there is no general definition of Quality Indicators (QIs) to measure PhC. Recognising this, a working group in a European association of PhC researchers, the Pharmaceutical Care Network Europe (PCNE), was established in 2020.

**Aim:**

This research aimed to review existing definitions of QIs and develop a definition of QIs for PhC.

**Method:**

A two-step procedure was applied. Firstly, a systematic literature review was conducted to identify existing QI definitions that were summarised. Secondly, an expert panel, comprised of 17 international experts from 14 countries, participated in two surveys and a discussion using a modified Delphi technique to develop the definition of QIs for PhC.

**Results:**

A total of 182 QI definitions were identified from 174 articles. Of these, 63 QI definitions (35%) cited one of five references as the source. Sixteen aspects that construct QI definitions were derived from the identified definitions. As a result of the Delphi study, the panel reached an agreement on a one-sentence definition of QIs for PhC: “quality indicators for pharmaceutical care are validated measurement tools to monitor structures, processes or outcomes in the context of care provided by pharmacists”.

**Conclusion:**

Building upon existing definition of QIs, an international expert panel developed the PCNE definition of QIs for PhC. This definition is intended for universal use amongst researchers and healthcare providers in PhC.

**Supplementary Information:**

The online version contains supplementary material available at 10.1007/s11096-023-01631-8.

## Impact statements

- Considering the growing number of countries implementing quality indicators for pharmaceutical care, this internationally agreed definition of quality indicators for pharmaceutical care will support harmonisation of terminology in the literature.
- The findings of this study, derived from a systematic literature review and a modified Delphi study, will contribute to a deeper understanding of quality measurement in pharmaceutical care.
- These findings will also provide a foundation for the development of a more unified and effective approach to monitoring pharmaceutical care quality.

## Introduction

The role of pharmacists in providing pharmaceutical care (PhC) has become well established over the past 40 years. PhC is defined as the 'pharmacist’s contribution to the care of individuals in order to optimise medicines use and improve health outcomes' [1]. Given the health concerns and the economic burden associated with medication errors, achieving high quality care for patients and the community underpinned by an evidence-based approach has become increasingly important worldwide. A widely used method to measure quality of care is the use of quality indicators (QIs). QIs are tools for monitoring the quality of care provided by healthcare professionals, promoting quality improvement activities, making comparisons over time between institutions or supporting consumers to choose healthcare providers [2]. Hence, QIs are recognised mechanism for evaluating the quality of care if they have been robustly developed and their measurement properties scientifically established. In the field of pharmacy practice, 15 countries have developed QIs: Argentina [3], Australia [4], Brazil [5], Canada [6], Germany [7], Japan [8–10], the Netherlands [11, 12], Spain [13], Sweden [14], Thailand [15], UK [16], USA [17], Ethiopia [18], Uganda [18] and Zimbabwe [18].

Despite its importance, no universally accepted definition of QIs exists in PhC research. A systematic literature review on QIs found that even though the role of measurement tools has the goal of quality improvement, about 20 name variations exist (e.g., QIs, clinical indicators or performance measures) [19]. The variety of QI definitions has made it challenging to establish a common understanding of measuring quality of care, resulting in confusion amongst healthcare providers and researchers.

Recognising this, a working group on guidelines and indicators was established (co-leads: KF and MT) in February 2020 in a European association of researchers in the field of PhC, the Pharmaceutical Care Network Europe (PCNE). PCNE was established in 1994 and became an official association in 2004. With around 50 institutional and 30 individual members from around the world, the aim of PCNE is to help to promote PhC delivered by pharmacists in Europe and elsewhere.

### Aim

The aims of this study were (1) to review existing definitions of QIs and (2) to develop PCNE definition of QIs for PhC.

### Ethics approval

An ethics approval was not required for this study as it was conducted as an internal working group activity within PCNE.

## Method

A two-step procedure was applied: a systematic literature review of existing QI definitions and international consensus development of the definition of QIs for PhC.

### Literature review

The literature review was performed in accordance with the Preferred Reporting Items for Systematic Reviews and Meta-Analyses statement [20]. The literature review aimed to identify existing QI definitions in healthcare. In this study, the term "aspects of QI definition" was operationally defined as the distinct elements derived from various QI definitions in the literature, which collectively constitute a comprehensive understanding of QIs. Given the lengths of a one-sentence definition of QIs could be different depending on the number of aspects included in the definition, the following five research questions were addressed: (1) how many QI definitions exist in literature in the field of healthcare? (2) what is the most commonly cited QI definition? (3) what are common aspects of QI definitions? (4) how many aspects are included per QI definition? and (5) what terms are used to describe each aspect of QI definitions?

First, CINAHL, EMBASE, Global Health, International Pharmaceutical Abstract, MEDLINE, PubMed and Web of Science databases were searched up to March 2020. No restriction on year of study was applied. Exact search dates for each database with the search strategies are included in supplementary material A. Articles were included if they fulfilled the following criteria: (A) the article was peer-reviewed and published in English and (B) the publication contained a definition of QIs. Second, an internet search using Google was conducted to capture additional QI definitions listed in the websites of relevant organisations responsible for quality improvement. Potentially relevant organisation’s websites, found in the process of literature review, were also searched.

The retrieved articles were transferred into Endnote to remove duplicates. Then initial screening of journal names, titles and abstracts was conducted by one author (KF) to remove irrelevant articles. Because this review necessitated screening of many articles to ascertain whether they contained a definition of QIs, QI definitions were programmatically identified and extracted from retrieved articles using Python 3.6.5 (Python Software Foundation, https://www.python.org/). To identify existing QI definitions from the literatures, we regarded a sentence as a definition of QIs when the sentence contained one of the pre-defined keywords (e.g. “indicator is”, “indicators are”, “QI is” or “QIs are”) which cited at least one reference. This process, programmatically carried out by looping through sentences within articles, greatly improved the efficiency of identifying QI definitions.

All references cited for a QI definition in each article were grouped by the year of publication and authors to identify an original source, if available. In addition, the retrieved definitions were analysed to identify key aspects of QI definitions. Candidate aspects of QI definitions were recognised by 25 international researchers from 11 countries during a PCNE workshop on 7th and 8th February 2020 in Egmond aan Zee, the Netherlands. The findings obtained from this literature review were used for the subsequent consensus development step.

### Consensus development

A modified Delphi technique was used to build consensus on the definition of QIs for PhC [21]. Twenty-nine experts were invited to the first round survey. Selection of the panel members was made by the two project leaders (KF and MT) from their professional networks, including participants of past PCNE workshops and individuals known for their experience and leadership roles in measuring healthcare quality. All participants in this modified Delphi process and the workshop participants in February 2020 agreed to participate in the research. The data were reported in aggregated format with comments de-identified.

#### Delphi—first round

The first round was carried out between November 2020 and January 2021 via an online platform, REDCap. The aim of the first round was to prioritise aspects of QIs for PhC that should be included in a QI definition for building a first version of the definition, and to identify issues that require further discussion. Participants were provided the key findings identified from the literature review to better understand existing QI definitions, and asked to (1) choose which term should be used to describe each aspect and (2) rate the necessity of each aspect for the definition of QIs using a 9-point scale from 1 = ‘definitely not necessary for a definition of QIs’ to 9 = ‘definitely necessary for a definition of QIs'. Participants were also asked to provide comments and additional aspects for a definition of QIs in free text. An aspect with a median score of 7–9 and percent agreement ≥ 80% was considered as ‘necessary’ for a definition of QIs. Any aspect which did not achieve consensus as ‘necessary’ was discussed at the subsequent expert panel meeting. Based on the first round results, KF and MT developed a first version of definition of QIs for PhC. This definition and the results of the first round were shared to participants by email.

#### Expert panel meeting

An online expert panel meeting was conducted on 1st October 2021. The aim of this meeting was to modify the first version of QI definition and discuss issues identified from the participants’ comments in the first round. Participants who had completed the first round were invited to this meeting. The discussion was audio-recorded and transcribed verbatim with additional notes taken by KF. A modified version of QI definition as well as a summary of discussion was shared to participants by email.

#### Delphi—second round

The second round was carried out between December 2021 and January 2022 using the same platform as the 1st round. Participants who had completed the first round were invited. The aim of the second round was to build consensus on the definition of QIs for PhC that were modified at the expert panel meeting. In this round, participants were given information about what modification was made from the first version of the QI definition and their reasons. Participants were asked to rate their level of agreement with each modification on a scale of 1–9, with 1 being “strongly disagree” and 9 being “strongly agree”. An answer with a median score of 7–9, without disagreement (i.e., fewer than four panellists gave a score of 1–3) was considered as “consensus reached” [21]. The results of the second round were sent to the participants, asking if any further modifications were required.

## Results

### Literature review

Question 1. How many QI definitions exist in the literature in the field of healthcare?

**Fig. 1:**
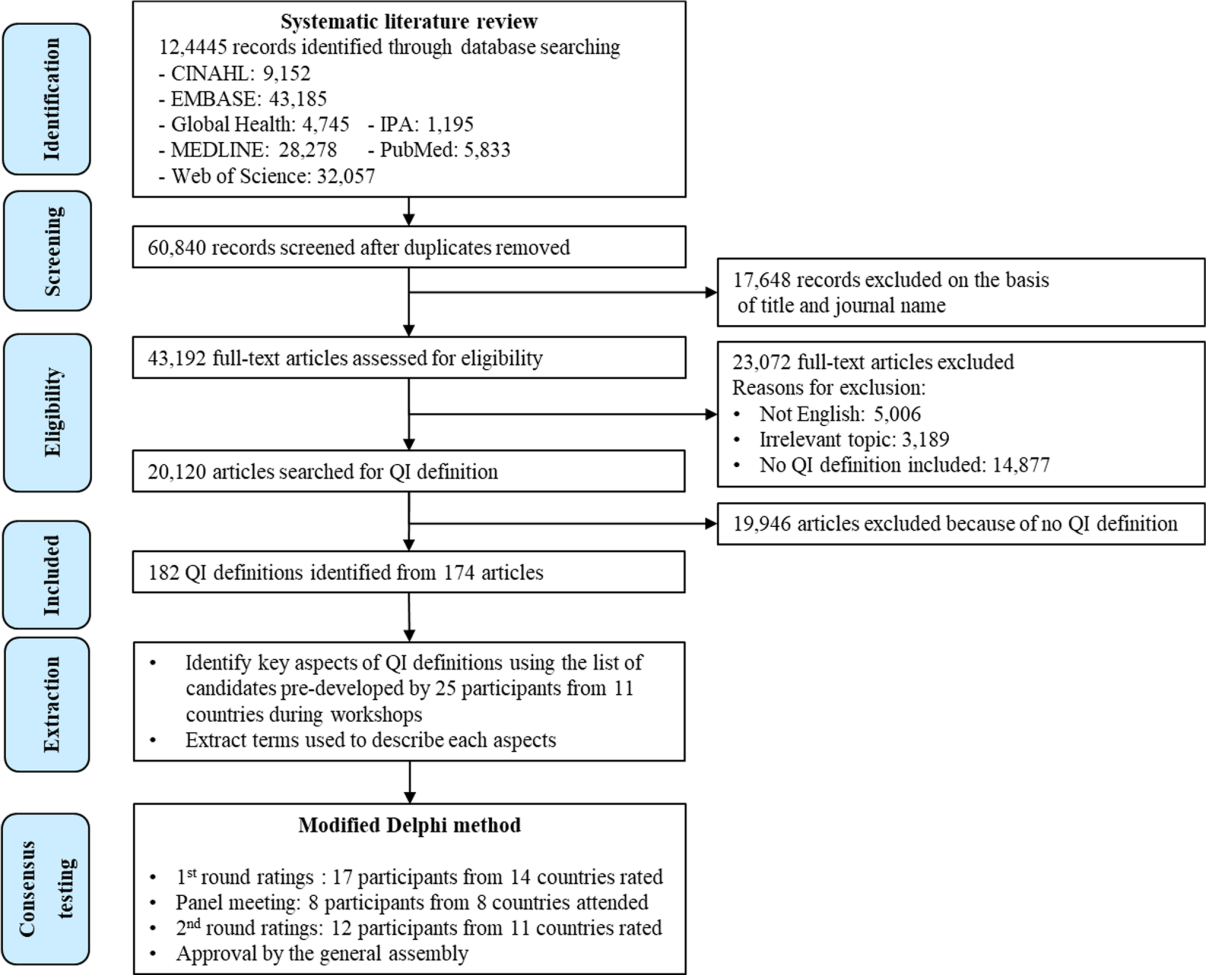
Study flow diagram

Initially, a total of 124,445 articles were obtained. The sample included 63,605 duplicate records, which were removed (Fig. 1). After the manual screening, 20,120 full texts were programmatically assessed to search for QI definitions. No additional QI definitions were identified via internet search. As a result, a total of 182 QI definitions were identified from 174 articles, which cited 101 different references for QI definitions.

Question 2. What is the most commonly cited QI definition?

Table 1 shows the five most frequently cited sources for QI definitions which accounted for 35% of all cited references (63/182). The source most frequently directly cited by these articles was Campbell et al. in 2003 [22], appearing in 18 articles. However, Campbell’s paper further cited an article published by McGlynn et al. in 1998 [23]. Similarly, Campbell et al. in 2002 [24], cited by 13 articles, referenced the QI definition proposed by Lawrence et al. in 1997 [25]. While the McGlynn’s article did not describe the specific sentence used in the Campbell’s article published in 2003, Lawrence et al. distinctly articulated the definition of QIs in their paper. Consequently, we concluded that, although indirectly, the QI definition by Lawrence et al. (1997) [25] was the most frequently cited.

**Table 1:** Five most commonly cited references for definitions of quality indicators (QIs)

References	Count (%) N = 182 articles	Definition	Citing reference
Campbell SM et al. [22]	18 (9.9)	Indicators are explicitly defined and measurable items referring to the structures, processes, or outcomes of care	McGlynn EA et al. (1998)
Campbell SM et al. [24]	13 (7.1)	A measurable element of practice performance for which there is evidence or consensus that it can be used to assess the quality, and hence change in the quality, of care provided	Lawrence M et al. (1997)
Mainz [2]	13 (7.1)	Indicators have been defined in several different ways: As measures that assess a particular health care process or outcome [a] As quantitative measures that can be used to monitor and evaluate the quality of important governance, management, clinical, and support functions that affect patient outcomes [b] As measurement tools, screens, or flags that are used as guides to monitor, evaluate, and improve the quality of patient care, clinical support services, and organizational function that affect patient outcomes [c]	a. Worning AM et al. [27] b. The joint commission on accreditation of healthcare organizations [28] c. Canadian council on health services accreditation [29]
Lawrence et al. [25]	12 (6.6)	A measurable element of practice performance for which there is evidence or consensus that it can be used to assess the quality, and hence change in the quality, of care provided	Original source
McGlynn et al. [23]	7 (3.8)	Not specifically defined	Original source

Question 3. What are common aspects of QI definitions?

Referring to the list of pre-developed candidates for aspects of QI definitions that were developed in the PCNE workshop in February 2020, the following 16 common aspects of QI definitions were derived from the identified QI definitions: name, item, domain, provider, subject, types of care, population, purpose, setting, measurement properties, characteristics, quality dimensions, users, unit of analysis/comparison, data sources and development methods (Table 2). Percentages of each aspect included in the 182 QI definitions varied depending on the aspect ranging from 1.1% (data sources) to 100% (name and item).

**Table 2:** Sixteen aspects of the definition of quality indicators (QIs)

Aspect	% of inclusion (n = 182)	Description	Selected term	Necessity (n = 17)
**Name**	**100**	**Terminology of tools**	**Quality indicators**	**9**
**Item**	**100**	**What are QIs?**	**Measures**	**8**
**Domain**	**13.2**	**Should Donabedian framework be included?**	**Yes**	**8**
**Provider**	**6.0**	**Who are the providers of care?**	**Pharmacists**	**7**
**Subject**	**61**	**What is evaluated by QIs?**	**Structures, processes, or outcomes of care**	**8**
**Types of care**	**N/A**	**What types of care is evaluated?**	**Pharmaceutical care**	**7**
**Population**	**9.3**	**Who is the target population?**	**Patients**	**7**
**Purpose**	**39.6**	**What are QIs used for?**	**Monitoring and improving quality of care**	**8**
Setting	2.2	Where is the care provided	Hospitals, primary care, community pharmacies	5
Measurement properties	45.1	What measurement properties should QIs have?	Evidence-based, measurable	6
**Characteristics**	**7.1**	**What are QIs’ characteristics?**	**Usually described with a denominator and a numerator** **described as rates or percentages**	**7**
Quality dimensions	4.4	Should quality domains defined by IOM/ WHO be included?	No	4
Users	6.0	Who are the users of QI scores?	Pharmacists, health care providers	5
Unit of analysis	7.7	What is the unit of analysis?	At the level of institutions, practitioners and patients	5
Data sources	1.1	What data sources are needed?	Usually derived from retrospective reviews of medical records	5
**Development methods**	**2.2**	**How are QIs developed?**	**Developed based on expert consensus methods, developed based on literature review**	**9**

"% of inclusion" represents the percentage of QI definitions identified in our literature review which included each aspect (N = 182 definitions). "Description" indicates the question asked in relation to each aspect. "Selected term" includes the specific term(s) chosen by the panellists for each aspect. "Necessity" indicates the median score by the 17 panellists who rated each aspect. A row highlighted with bold letters shows that the corresponding aspect was considered as ‘necessary’ for a definition of QIs. N/A: not applicable

Question 4. How many aspects are included per QI definition?

The median number of aspects included per QI definition was 4.0 (min 2, max 7). Moreover, of 182 QI definitions, 46 definitions (25%) defined the measurement tool as singular (e.g., indicator or quality measure) whilst the rest of 136 (75%) definitions as plural (e.g., indicators or quality measures).

Question 5. What terms are used to describe each aspect of QI definitions?

Terms used to describe each aspect of QIs varied. These identified terms were used as answer options for the subsequent consensus development. Detailed descriptions of these terms can be found in Supplementary material B.

### Consensus development

#### Delphi—first round

Seventeen participants from 14 countries (i.e., Australia, Belgium, Germany, Ireland, Japan, Jordan, Netherlands, Norway, Portugal, Slovenia, Spain, Sweden, UK, USA) completed the first round survey (response rate 59%). Ten of the 16 aspects of QI definitions were assessed as “necessary” for the QI definition (Table 2). The panel also agreed that the form of the measurement tool should be plural rather than singular. Using the selected terms for the 10 aspects, a first version of QI definition was developed by KF and MT as follows: Quality indicators to monitor and improve pharmaceutical care are consensus-based measures of structures and processes in the pharmacies and of outcomes in patient populations, usually described by a denominator and a numerator. In addition, several issues were identified based on comments documented by the participants, which were discussed at the subsequent expert panel meeting.

#### Expert panel meeting

In addition to the two co-leaders, eight of the 17 participants attended an online discussion. The following eight points were discussed (Table 3):

**Table 3:** Summary of discussion in the expert panel meeting held between the two Delphi rounds

Aspect and key discussion point	Quote	Decision
1. Characteristics: Is it necessary to include the 'characteristics' aspect: 'usually described by a denominator and a numerator' as this does not apply to the measurement of structure indicators?	It's a good idea to keep a definition as simple as possible without losing details. So the characteristics here, they are important but not actually needed in the definition	Deletion
2. Development method: Should we include not only 'consensus-based' but also 'based on literature review' in the QI definition?	Consensus-based’ measures is just one way to arrive at the QI. The notion of having exclusively ‘consensus-based’ measure in the definition might not be appropriate in every case. Given evidence could be created by expert consensus and literature review, ‘evidence-based’ is probably a better term than ‘consensus-based Strong level of evidence is not always available for QIs, especially for structure indicators. In addition, evidence can change all the time. ‘Validated’ measures is probably a better term than ‘evidence-based’ measures Evidence-based’ is connected to a guideline or a therapeutic option while ‘validated’ is the term which links to a measurement tool, like a survey, measurement of adherence, or a measure of quality of life. You can have a face-validated structure indicator, which is a very low level of validation and all the way through to QIs with high predictive validity	‘Consensus-based’ should be replaced with ‘validated’
3. Name and types of care: Should the definition start with “Quality indicators for pharmaceutical care”?	Without stating specific care, the definition of QIs would be ambiguous When we start the definition with “quality indicators for pharmaceutical care”, maybe that can also be a solution because quality indicators are a lot of more than just pharmaceutical care, or also quality indicators in GPs and another domains	Focus this QI definition only on pharmaceutical care
4. Providers: Who are the providers of pharmaceutical care?	In the PCNE definition of Pharmaceutical care, Pharmaceutical care is defined as the contribution of the 'pharmacist', which implies collaboration between different contributors and does not exclude any other healthcare provider such as technicians. In addition, we strongly feel that as medical care is the medical doctors’ care, there also needs to be something to show what pharmacists do	Specify 'pharmacist' as the provider
5. Population: Who is the target population? (e.g., patients, individuals, persons)	Pharmaceutical care is not just provided to people who were sick. Care could be for maintaining wellness, screening or prevention. In addition, a patient with high blood pressure doesn't consider themselves a patient. All of the time they consider themselves an individual even when they're sick Given the PCNE definition of pharmaceutical care has an ‘individuals’ rather than ‘patients’ nor ‘persons’, ‘individuals’ is a better word The PCNE definition of pharmaceutical care already states target population as “individuals”, this aspect can skip from the definition	Remove target population from the definition
6. Measurement tools: Are the indicators actually measurement tools?	Measures is the data you get through using quality indicators. QIs are probably measurement tools. I agree in part with the way you have discerned the difference between measurement tools and measures	‘Measures' should be replaced with 'measurement tools
7. Settings: Where is the care provided?	Pharmaceutical care may be delivered not in the pharmacy but in the clinic or outside of the room or discharge in hospital. It's not restricted to in the pharmacies	Remove the term ‘in the pharmacies’ from the definition
8. Purposes	How I understand it is that QI score could go both ways. So, it's actually more monitoring than improving. So, the idea behind quality indicators is of course to improve. However, quality can also decrease. So, in that way, we are ‘monitoring’ quality of care using indicators	The term ‘improve’ should be removed from the definition

*Characteristics*: Is it necessary to include the 'characteristics' aspect: 'usually described by a denominator and a numerator' as this does not apply to the measurement of structure indicators?

Although this characteristic is applicable, the panel agreed that our QI definition should be as simple as possible without losing details. Therefore, the panel decided to delete this aspect from the definition.

*Development method*: Should we include not only 'consensus-based' but also 'based on literature review' in the QI definition?

Given building consensus is one of the methods for validating QIs, the panel decided to replace “consensus-based” with “validated”.

*Name and types of care*: Should the definition start with “Quality indicators for pharmaceutical care”?

QIs are used to evaluate quality of different types of care, such as medical care or nursing care. Without stating specific care, the definition of QIs would be ambiguous. Accordingly, the panel decided to focus this QI definition only on PhC by starting the definition with “Quality indicators for pharmaceutical care”.

*Providers*: Who are the providers of PhC?

Pharmaceutical care was defined as the contribution of the 'pharmacist' [1], which implies collaboration between different contributors and does not exclude any other healthcare provider (e.g., pharmacy technicians). Accordingly, the panel recognised the importance of clearly specifying ‘pharmacists’ as a provider of PhC in the definition.

*Population*: Who is the target population? (e.g., patients, individuals, persons).

Some panellists felt that PhC is provided not only to people who were sick but also to people in good health for maintaining wellness, screening or prevention. Considering that QIs for PhC could evaluate quality of care provided to healthy people, the panel considered that ‘individuals’ is a better term rather than ‘patient’. However, given that the PCNE definition of PhC already states target population as “individuals” [1], this aspect was skipped from the definition.

*Measurement tools*: Are the indicators actually measurement tools?

One panellist emphasised the importance of differentiating measures and measurement tools, by saying that “measures is the data you get through using quality indicators. QIs are probably measurement tools” (Table 3). The panel determined to replace ‘measures’ with ‘measurement tools’ in the definition.

*Settings*: Where is the care provided?

One panellist stated that settings of providing PhC should not be restricted to pharmacies because PhC may also be delivered in a variety of settings outside of pharmacy such as clinics, hospitals, nursing homes (Table 3). The panel agreed that as long as types of care (i.e., PhC) and providers (i.e., pharmacists) are included in the definition, the definition would not need to specify settings where the care is provided.

*Purposes*: What are QIs used for?

The panel agreed that the purposes of using QIs are different depending on who is the user. For example, citizens could use QIs for choosing their preferred facilities, the government could use QIs for pay for performance initiatives, and healthcare providers could use QIs for improving quality of care provided.

However, monitoring quality of care using QIs is the basis of aiming to further purposes. Therefore, the panel decided to remove ‘improve’ from the definition and only state ‘monitor’ as a purpose of using QIs.

As a result, the 1st version of the QI definition was updated as follows: Quality indicators for pharmaceutical care are validated measurement tools to monitor structures, processes or outcomes in the context of care provided by pharmacists.

#### Delphi—second round

Twelve of 17 members who had completed the first round answered the second round survey. The details of the second round results are shown in Supplementary material C. An agreement of the definition of QIs for PhC was reached without any modification. The final version was presented to the general assembly of PCNE on 11th February 2022 in Lisbon, Portugal. The general assembly approved it unanimously. As a result, the proposed definition became the official PCNE definition of QIs for PhC.

## Discussion

Agreement on the definition of QIs for PhC was reached following a systematic literature review of the literature and a modified Delphi study. The agreed definition, “quality indicators for pharmaceutical care are validated measurement tools to monitor structures, processes or outcomes in the context of care provided by pharmacists”, consists of seven aspects of QI definitions. The aspects included in the definition were: (1) name: terminology of the tool is “quality indicators”, (2) subject: QIs evaluate quality of “pharmaceutical care”, (3) measurement properties: QIs should be “validated”, (4) item: QIs are “measurement tools”, (5) purpose: purpose of using QIs is to “monitor” quality of care, (6) domain: QIs evaluate all three domains defined by Donabedian (i.e., structures, processes or outcomes of care) [26], (7) provider: QIs for PhC evaluated quality of care provided by “pharmacists”. This definition officially accepted by the PCNE is intended to be used universally amongst researchers and healthcare providers in PhC.

The systematic literature review identified many different aspects of QIs and those different definitions contained some but not most of these aspects. This emphasised the importance of comprehensively identifying the different aspects of QI definitions, and evaluating the necessity of each aspect for the definition of QIs for PhC. In this regard, this review process avoided missing important aspects that were included in existing QI definitions. The most commonly cited QI definition, as defined by Lawrence et al. [25], focused on process of care provided, which makes us realise the importance of redefining QIs for PhC.

Using the study findings identified from the literature review, the first round of the Delphi process prioritised 10 aspects and determined specific terms which should be included in the definition of QIs for PhC. The subsequent online discussion resulted in reducing the number of aspects from 10 to seven and rephrasing the definition statement. The expert panel agreed to specify pharmacists as the provider of PhC in the definition of QIs according to the understanding of PCNE and its definition issued in 2013 [1].

We acknowledge that our approach has some limitations. First, the systematic literature review was conducted by one author applying a programmatic approach to the identification of QI definitions. Some definitions of QI may have been missed. Second, not all participants who had completed the first round participated in the subsequent online discussion and the second round. However, because 12 people from 11 countries participated in the two rounds of surveys, and a summary of the expert panel meeting was shared to those who had completed the first round, important views were unlikely to have been overlooked. Third, our study was limited by a lack of geographical diversity in the expert panel. Given that experts from South America and Africa were not included in this study, this study might have overlooked region-specific perspectives and nuances.

## Conclusion

Referring to existing definition of QIs, an international expert panel developed the PCNE definition of QIs for PhC through a consensus building process. It reads “quality indicators for pharmaceutical care are validated measurement tools to monitor structures, processes or outcomes in the context of care provided by pharmacists”. This definition is intended to be used universally amongst researchers and healthcare providers in PhC. With the established definition of QIs for PhC, the next step should be the widespread communication and dissemination of this definition across the PhC community. This shared understanding of QIs for PhC will guide future development and implementation of internationally applicable QIs that assist healthcare providers in monitoring and improving the quality of their services.

### Supplementary Information

Below is the link to the electronic supplementary material.

Supplementary file1 (PDF 118 kb)

Supplementary file2 (PDF 165 kb)

Supplementary file3 (PDF 930 kb)
