# Supplementary material for: Pharmaceutical Care Network Europe definition of quality indicators for pharmaceutical care: a systematic literature review and international consensus development
Source: Int J Clin Pharm. 2023 Aug 30;46(1):70–9. doi: 10.1007/s11096-023-01631-8 (PMC10830737; doi:10.1007/s11096-023-01631-8)
Supplement: Supplementary file 1 — Supplementary file1 (PDF 118 kb) [file 11096_2023_1631_MOESM1_ESM.pdf]

Supplementary material A. Search history

2020/04/11
Database: Ovid MEDLINE(R) ALL <1946 to April 09, 2020>

Search Strategy:
1 (quality adj3 indicator$).ti,ab. (12901)
2 (evidence based adj1 indicator$).ti,ab. (62)
3 "clinical indicator$".ti,ab. (3491)
4 "performance indicator$".ti,ab. (3615)
5 "prescribing indicator$".ti,ab. (186)
6 "medication related indicator$".ti,ab. (3)
7 "safety indicator$".ti,ab. (691)
8 "NICE indicator$".ti,ab. (1)
9 "practice level indicator$".ti,ab. (2)
10 "structure indicator$".ti,ab. (81)
11 "process indicator$".ti,ab. (858)
12 "outcome indicator$".ti,ab. (1615)
13 (indicator$ adj1 assess*).ti,ab. (585)
14 (indicator$ adj5 prescr*).ti,ab. (692)
15 "quality measure$".ti,ab. (7352)
16 "performance measure$".ti,ab. (10880)
17 "core measure$".ti,ab. (575)
18 "quality metrics".ti,ab. (1890)
19 "review criteria".ti,ab. (705)
20 1 or 2 or 3 or 4 or 5 or 6 or 7 or 8 or 9 or 10 or 11 or 12 or 13 or 14 or 15 or 16 or 17 or 18 or 19 (42561)
21 patient$.ti,ab. (6577658)
22 care.ti,ab. (1362863)
23 health*.ti,ab. (2659699)
24 21 or 22 or 23 (8600564)
25 20 and 24 (28278)

2020/04/11
Database: International Pharmaceutical Abstracts <1970 to March 2020>

Search Strategy:
1 (quality adj3 indicator$).ti,ab. (404)
2 (evidence based adj1 indicator$).ti,ab. (3)
3 "clinical indicator$".ti,ab. (84)
4 "performance indicator$".ti,ab. (122)
5 "prescribing indicator$".ti,ab. (41)
6 "medication related indicator$".ti,ab. (0)
7 "safety indicator$".ti,ab. (28)
8 "NICE indicator$".ti,ab. (0)
9 "practice level indicator$".ti,ab. (1)
10 "structure indicator$".ti,ab. (3)
11 "process indicator$".ti,ab. (42)
12 "outcome indicator$".ti,ab. (72)

13 (indicator$ adj1 assess*).ti,ab. (9)
14 (indicator$ adj5 prescr*).ti,ab. (122)
15 "quality measure$".ti,ab. (274)
16 "performance measure$".ti,ab. (312)
17 "core measure$".ti,ab. (121)
18 "quality metrics".ti,ab. (37)
19 "review criteria".ti,ab. (40)
20 1 or 2 or 3 or 4 or 5 or 6 or 7 or 8 or 9 or 10 or 11 or 12 or 13 or 14 or 15 or 16 or 17 or 18 or 19 (1462)
21 patient$.ti,ab. (218916)
22 care.ti,ab. (52786)
23 health*.ti,ab. (75400)
24 21 or 22 or 23 (270386)
25 20 and 24 (1195)

2020/04/11
Database: Embase Classic <1947 to 1973>, Embase <1974 to 2020 April 09>

Search Strategy:
1 (quality adj3 indicator$).ti,ab. (19050)
2 (evidence based adj1 indicator$).ti,ab. (79)
3 "clinical indicator$".ti,ab. (4974)
4 "performance indicator$".ti,ab. (5254)
5 "prescribing indicator$".ti,ab. (376)
6 "medication related indicator$".ti,ab. (4)
7 "safety indicator$".ti,ab. (963)
8 "NICE indicator$".ti,ab. (1)
9 "practice level indicator$".ti,ab. (2)
10 "structure indicator$".ti,ab. (112)
11 "process indicator$".ti,ab. (1114)
12 "outcome indicator$".ti,ab. (2159)
13 (indicator$ adj1 assess*).ti,ab. (783)
14 (indicator$ adj5 prescr*).ti,ab. (1148)
15 "quality measure$".ti,ab. (10636)
16 "performance measure$".ti,ab. (14179)
17 "core measure$".ti,ab. (992)
18 "quality metrics".ti,ab. (3267)
19 "review criteria".ti,ab. (880)
20 1 or 2 or 3 or 4 or 5 or 6 or 7 or 8 or 9 or 10 or 11 or 12 or 13 or 14 or 15 or 16 or 17 or 18 or 19 (60425)
21 patient$.ti,ab. (9917365)
22 care.ti,ab. (1887666)
23 health*.ti,ab. (3585647)
24 21 or 22 or 23 (12373170)
25 20 and 24 (43185)

2020/04/11
Database: Global Health <1910 to 2020 Week 13>

Search Strategy:
1 (quality adj3 indicator$).ti,ab. (3211)
2 (evidence based adj1 indicator$).ti,ab. (9)
3 "clinical indicator$".ti,ab. (563)
4 "performance indicator$".ti,ab. (899)
5 "prescribing indicator$".ti,ab. (84)
6 "medication related indicator$".ti,ab. (0)
7 "safety indicator$".ti,ab. (157)
8 "NICE indicator$".ti,ab. (0)
9 "practice level indicator$".ti,ab. (1)
10 "structure indicator$".ti,ab. (19)
11 "process indicator$".ti,ab. (272)
12 "outcome indicator$".ti,ab. (408)
13 (indicator$ adj1 assess*).ti,ab. (196)
14 (indicator$ adj5 prescr*).ti,ab. (205)
15 "quality measure$".ti,ab. (1020)
16 "performance measure$".ti,ab. (985)
17 "core measure$".ti,ab. (46)
18 "quality metrics".ti,ab. (98)
19 "review criteria".ti,ab. (99)
20 1 or 2 or 3 or 4 or 5 or 6 or 7 or 8 or 9 or 10 or 11 or 12 or 13 or 14 or 15 or 16 or 17 or 18 or 19 (7718)
21 patient$.ti,ab. (806350)
22 care.ti,ab. (224650)
23 health*.ti,ab. (794299)
24 21 or 22 or 23 (1465974)
25 20 and 24 (4745)

2020/04/11
Database: CINAHL

Search Strategy:
S26 S21 AND S25 (9,152)
S25 S22 OR S23 OR S24 (2,753,651)
S24 TI health* OR AB health* (1,275,928)
S23 TI care OR AB care (890,570)
S22 TI patient$ OR AB patient$ (1,572,060)
S21 S1 OR S2 OR S3 OR S4 OR S5 OR S6 OR S7 OR S8 OR S9 OR S10 OR S11 OR S12 OR S13 OR S14 OR S15 OR S16 OR S17 OR S18 OR S19 OR S20 (11,499)
S20 TI "review criteria" OR AB "review criteria" (392)
S19 TI "quality metrics" OR AB "quality metrics" (820)
S18 TI "core measure$" OR AB "core measure$" (198)
S17 TI "performance measure$" OR AB "performance measure$" (1,202)
S16 TI "quality measure$" OR AB "quality measure$" (744)
S15 TI indicator$ n5 prescr* OR AB indicator$ n5 prescr* (333)
S14 TI indicator$ n1 assess* OR AB indicator$ n1 assess* (925)
S13 TI "outcome indicator$" OR AB "outcome indicator$" (181)
S12 TI "process indicator$" OR AB "process indicator$" (48)

S11 TI "structure indicator$" OR AB "structure indicator$" (9)
S10 TI practice n1 indicator$ OR AB practice n1 indicator$ (138)
S9 TI "NICE indicator$" OR AB "NICE indicator$" (2)
S8 TI "safety indicator$" OR AB "safety indicator$" (121)
S7 TI medication n1 indicator$ OR AB medication n1 indicator$ (64)
S6 TI "medication related indicator$" OR AB "medication related indicator$" (0)
S5 TI "prescribing indicator$" OR AB "prescribing indicator$" (8)
S4 TI "performance indicator$" OR AB "performance indicator$" (302)
S3 TI "clinical indicator$" OR AB "clinical indicator$" (309)
S2 TI "evidence based" n1 indicator$ OR AB "evidence based" n1 indicator$ (91)
S1 TI quality n3 indicator$ OR AB quality n3 indicator$ (6,344)

2020/04/11
Database: Pubmed

Search Strategy:
Search Query Items found

#25 Search ((((((((((((((((((("quality indicator$"[Title/Abstract]) OR "quality of care indicator$"[Title/Abstract]) OR "evidence based indicator$"[Title/Abstract]) OR "clinical indicator$"[Title/Abstract]) OR "performance indicator$"[Title/Abstract]) OR "prescribing indicator$"[Title/Abstract]) OR "medication related indicator$"[Title/Abstract]) OR "medication-related indicator$"[Title/Abstract]) OR "safety indicator$"[Title/Abstract]) OR "NICE indicator$"[Title/Abstract]) OR "practice-level indicator$"[Title/Abstract]) OR "structure indicator$"[Title/Abstract]) OR "process indicator$"[Title/Abstract]) OR "outcome indicator$"[Title/Abstract]) OR "quality measure$"[Title/Abstract]) OR "performance measure$"[Title/Abstract]) OR "core measure$"[Title/Abstract]) OR "quality metrics"[Title/Abstract]) OR "review criteria"[Title/Abstract])) AND (((patient$[Title/Abstract]) OR care[Title/Abstract]) OR health*[Title/Abstract]) (5833)

#24 Search ((patient$[Title/Abstract]) OR care[Title/Abstract]) OR health*[Title/Abstract] (4462056)

#23 Search health*[Title/Abstract] (1998745)

#22 Search care[Title/Abstract] (1402428)

#21 Search patient$[Title/Abstract] (2193375)

#20 Search (((((((((((((((((("quality indicator$"[Title/Abstract]) OR "quality of care indicator$"[Title/Abstract]) OR "evidence based indicator$"[Title/Abstract]) OR "clinical indicator$"[Title/Abstract]) OR "performance indicator$"[Title/Abstract]) OR "prescribing indicator$"[Title/Abstract]) OR "medication related indicator$"[Title/Abstract]) OR "medication-related indicator$"[Title/Abstract]) OR "safety indicator$"[Title/Abstract]) OR "NICE indicator$"[Title/Abstract]) OR "practice-level indicator$"[Title/Abstract]) OR "structure indicator$"[Title/Abstract]) OR "process indicator$"[Title/Abstract]) OR "outcome indicator$"[Title/Abstract]) OR "quality measure$"[Title/Abstract]) OR "performance measure$"[Title/Abstract]) OR "core measure$"[Title/Abstract]) OR "quality metrics"[Title/Abstract]) OR "review criteria"[Title/Abstract] (10016)

#19 Search "review criteria"[Title/Abstract] (687)

#18 Search "quality metrics"[Title/Abstract] (1935)

#17 Search "core measure$"[Title/Abstract] (203)

#16 Search "performance measure$"[Title/Abstract] (1831)

#15 Search "quality measure$"[Title/Abstract] (1264)

#14 Search "outcome indicator$"[Title/Abstract] (336)

#13 Search "process indicator$"[Title/Abstract] (107)
#12 Search "structure indicator$"[Title/Abstract] (14)
#11 Search "practice-level indicator$"[Title/Abstract] (0)
#10 Search "NICE indicator$"[Title/Abstract] (0)
#9 Search "safety indicator$"[Title/Abstract] (204)
#8 Search "medication-related indicator$"[Title/Abstract] (0)
#7 Search "medication related indicator$"[Title/Abstract] (0)
#6 Search "prescribing indicator$"[Title/Abstract] (20)
#5 Search "performance indicator$"[Title/Abstract] (682)
#4 Search "clinical indicator$"[Title/Abstract](870)
#3 Search "evidence based indicator$"[Title/Abstract] (5)
#2 Search "quality of care indicator$"[Title/Abstract] (94)
#1 Search "quality indicator$"[Title/Abstract](2009)

2020/04/11
Database: Web of Science

Search Strategy:
Set Results
25 32,057 #24 AND #20
24 8,587,647 #23 OR #22 OR #21
23 3,039,170 TS=(health*)
22 1,511,446 TS=(care)
21 6,084,038 TS=(patient$)
20 103,768 #19 OR #18 OR #17 OR #16 OR #15 OR #14 OR #13 OR #12 OR #11 OR #10 OR #9 OR #8 OR #7 OR #6 OR #5 OR #4 OR #3 OR
#2 OR #1
19 726 TS=("review criteria")
18 5,475 TS=("quality metrics")
17 602 TS=("core measure$")
16 32,144 TS=("performance measure$")
15 10,648 TS=("quality measure$")
14 800 TS=(indicator$ NEAR/5 prescr*)
13 6,330 TS=(indicator$ NEAR/1 assess*)
12 1,563 TS=("outcome indicator$")
11 1,082 TS=("process indicator$")
10 209 TS=("structure indicator$")
9 2 TS=("practice level indicator$")
8 2 TS=("NICE indicator$")
7 1,104 TS=("safety indicator$")
6 4 TS=("medication related indicator$")
5 227 TS=("prescribing indicator$")
4 17,763 TS=("performance indicator$")
3 3,170 TS=("clinical indicator$")
2 178 TS=("evidence based" NEAR/1 indicator$)
1 27,966 TS=(quality NEAR/3 indicator$)
