# Supplementary material for: Pharmaceutical Care Network Europe definition of quality indicators for pharmaceutical care: a systematic literature review and international consensus development
Source: Int J Clin Pharm. 2023 Aug 30;46(1):70–9. doi: 10.1007/s11096-023-01631-8 (PMC10830737; doi:10.1007/s11096-023-01631-8)

Supplementary material B. 1st round results

Aspect1. Name: Terminology of measurement tools (multiple answers possible)

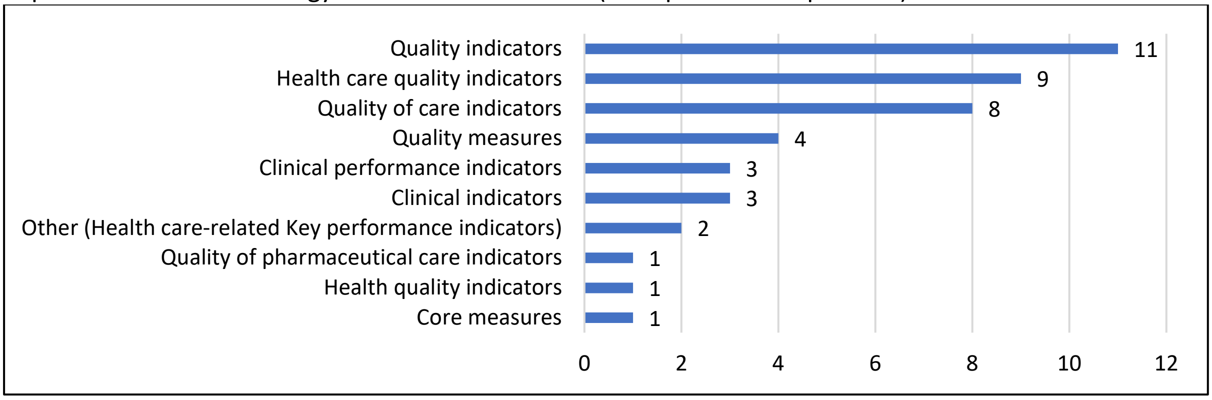

Aspect2. Item: What are QIs? (multiple answers possible)

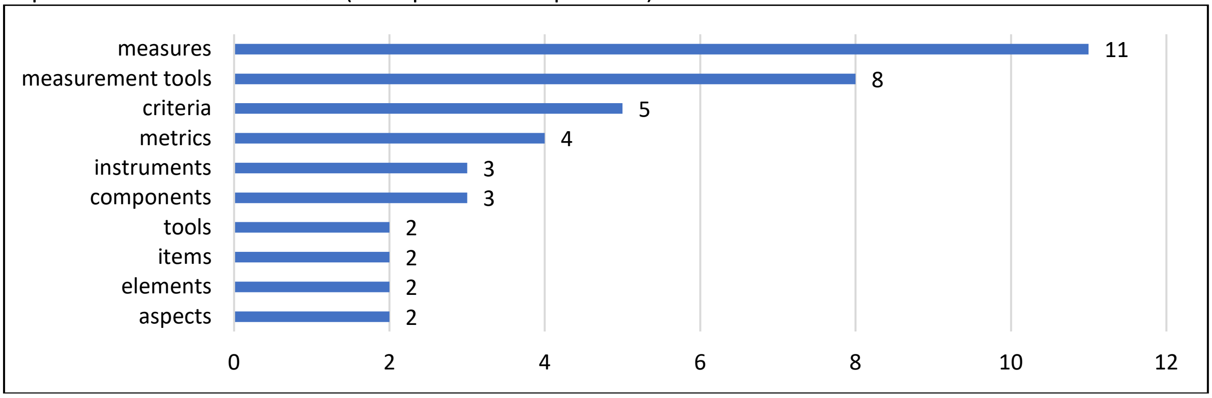

Aspect3. Domain:
Should the definition of the tool(s) include "structure, process, outcome" of Donabedian framework?

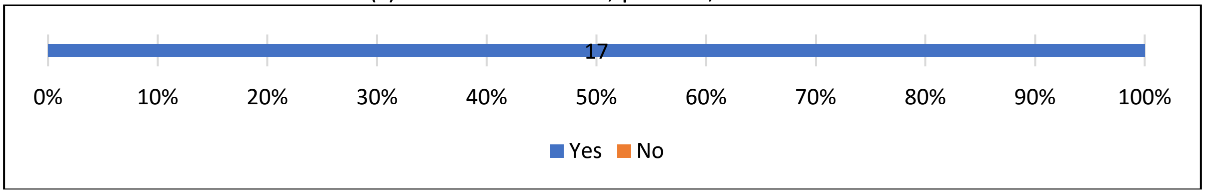

Aspect 4. Provider: Who are the providers of care? (multiple answers possible)

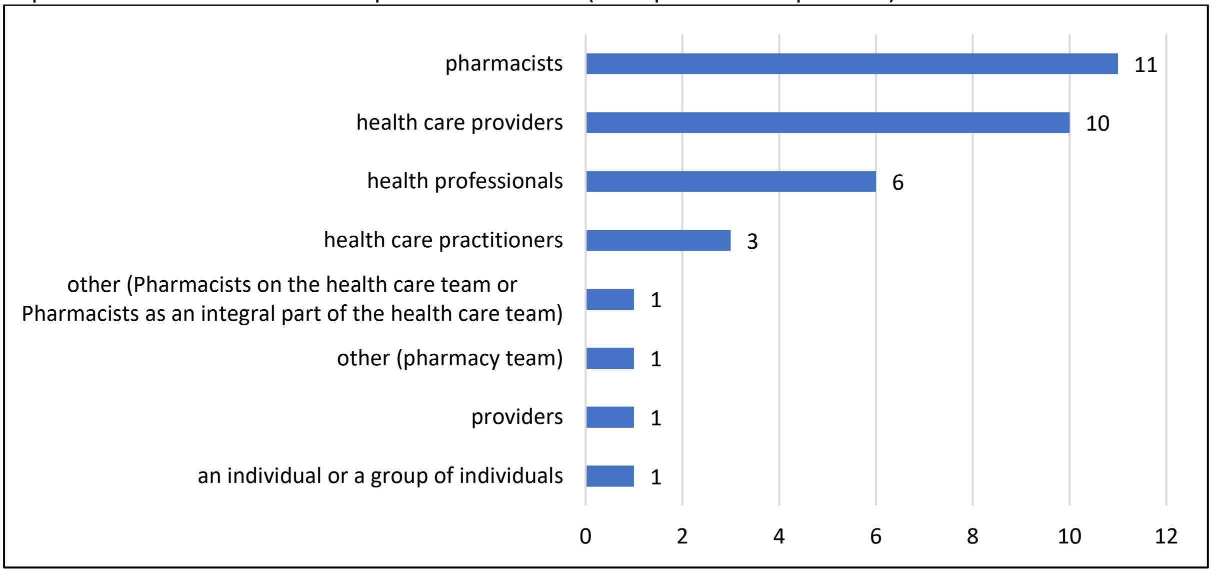

Aspect 5. Subject: What is evaluated by QIs? (multiple answers possible)

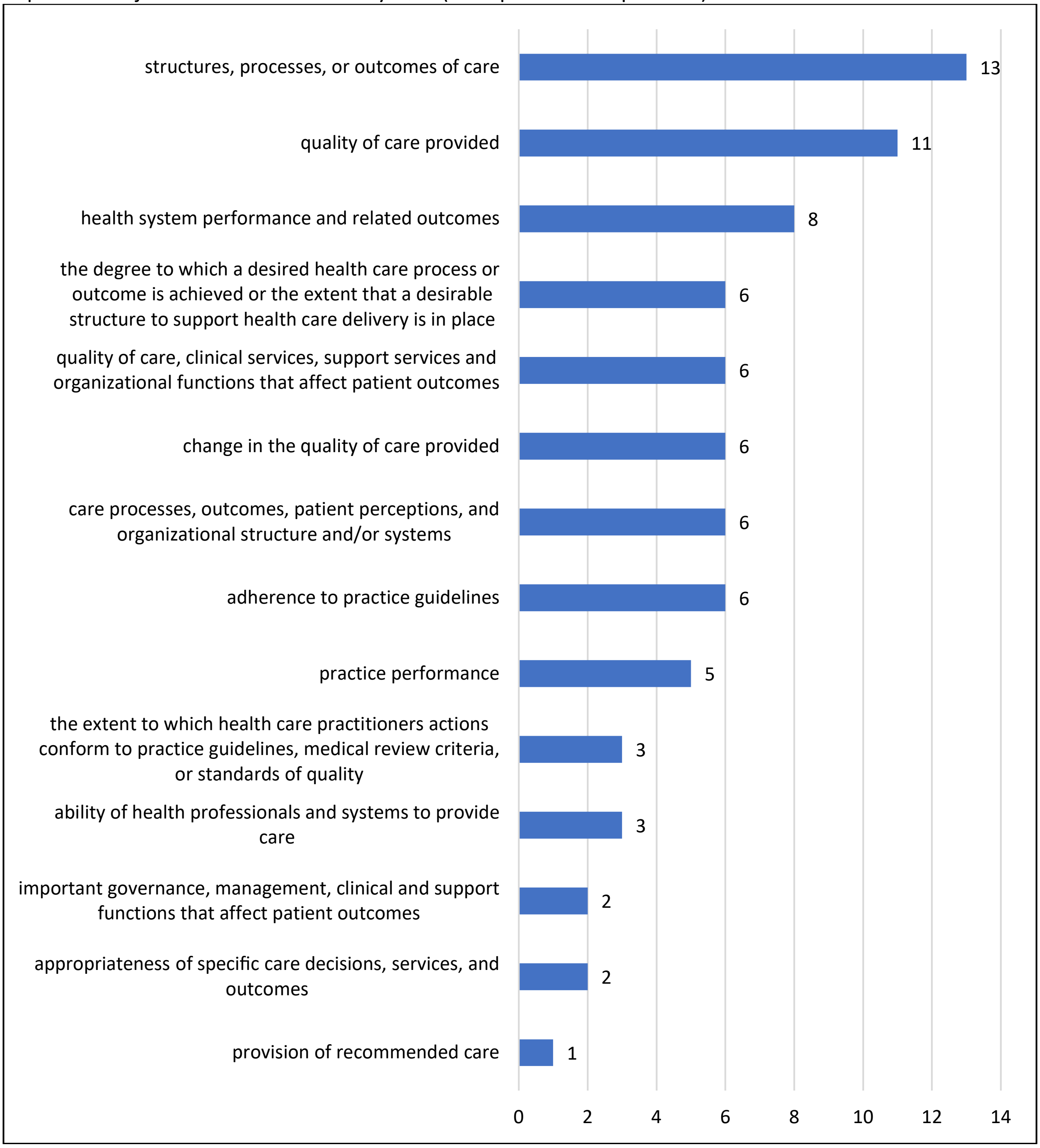

Aspect 6: Types of care: What types of care is evaluated? (multiple answers possible)

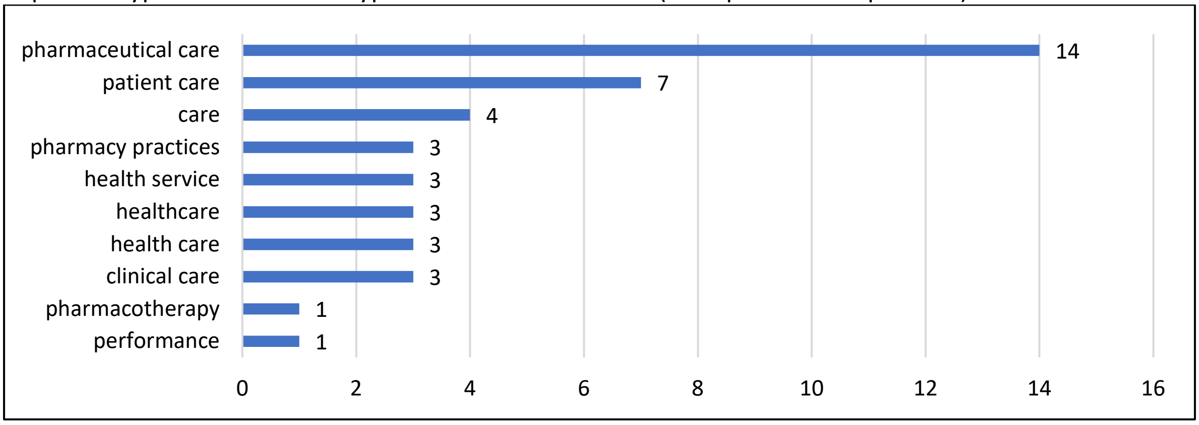

Aspect 7. Population: Who is the target population? (multiple answers possible)

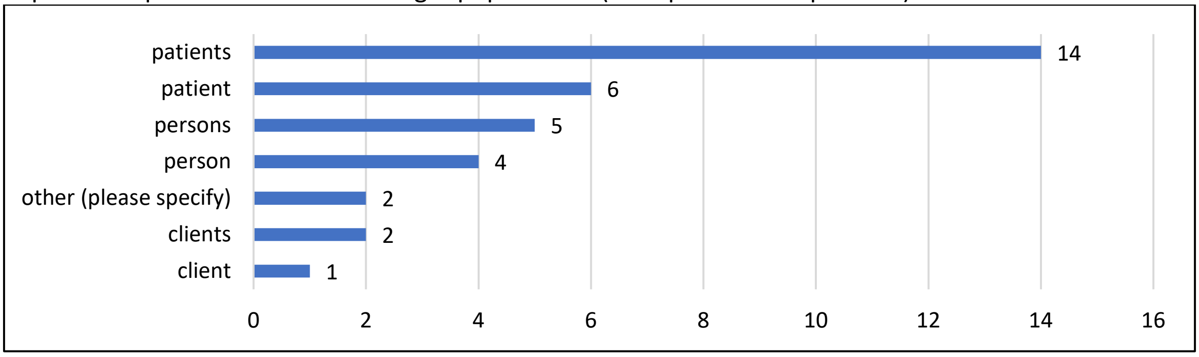

Aspect 8. Purpose: What are QIs used for? (multiple answers possible)

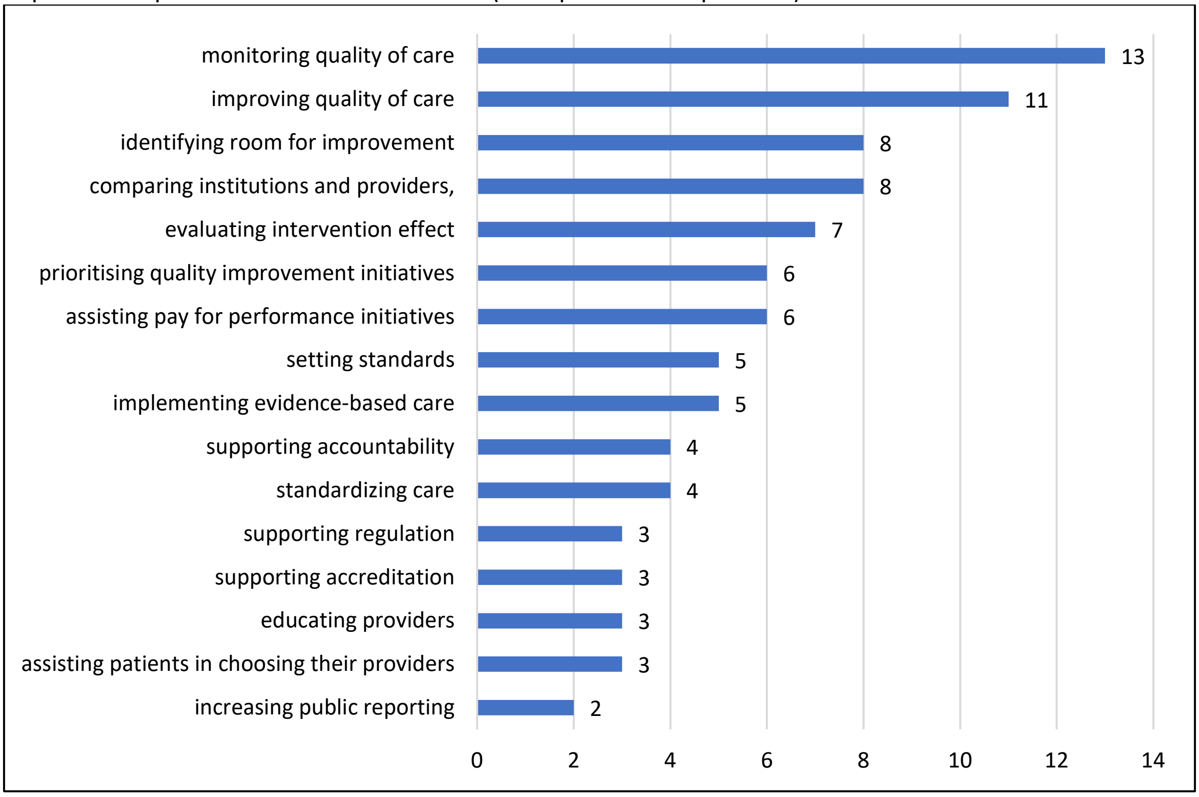

Aspect 9. Setting: Where is the care provided? (multiple answers possible)

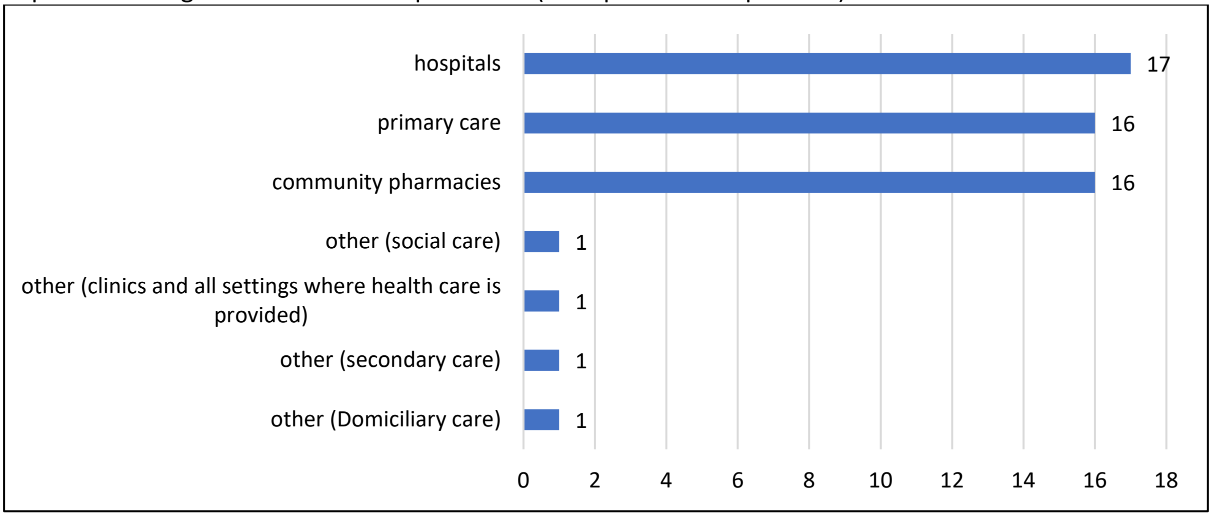

Aspect 10. Measurement properties: What measurement properties should QIs have? (multiple answers possible)

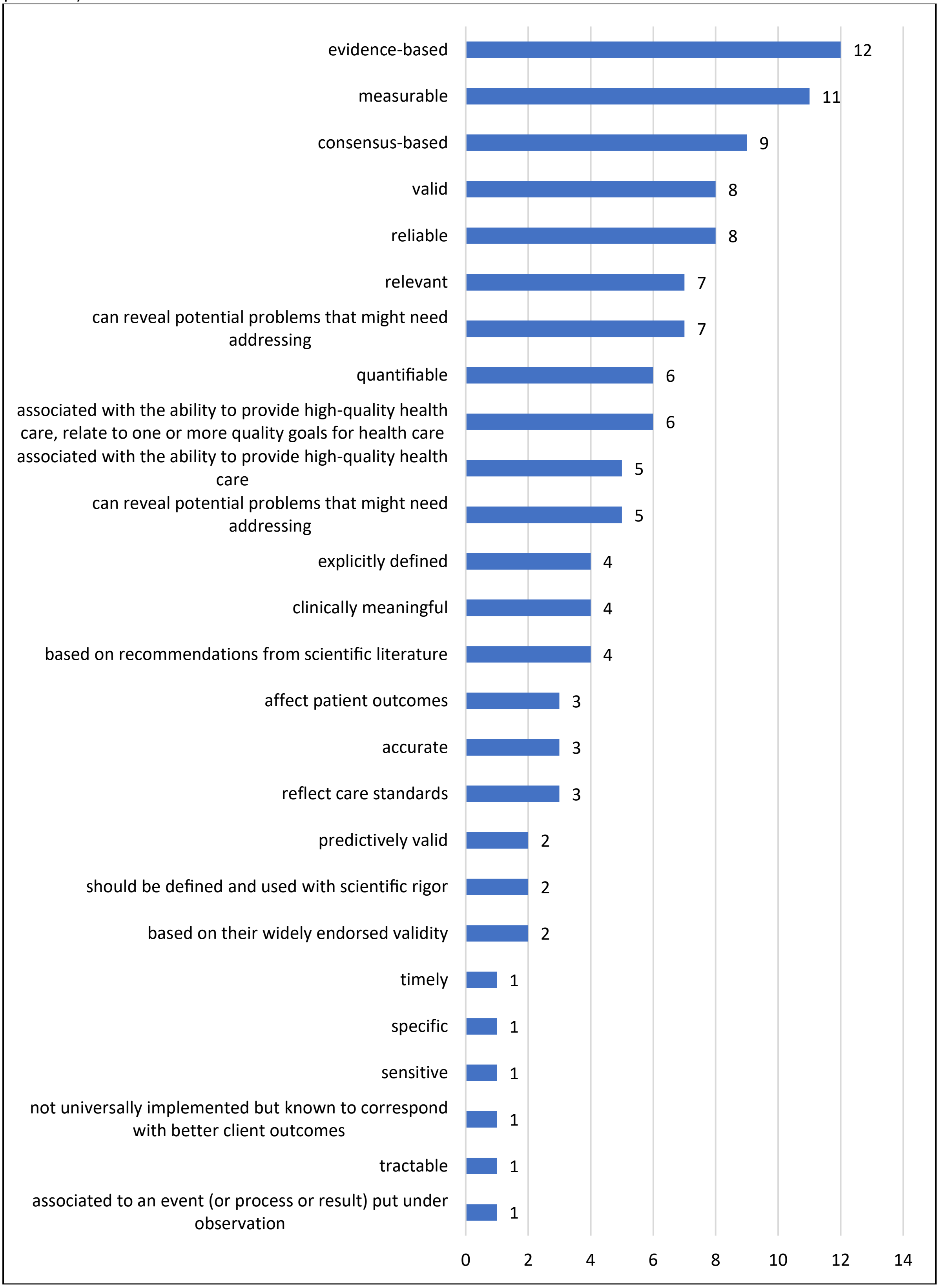

Aspect 11. Characteristics: What are QIs' characteristics? (multiple answers possible)

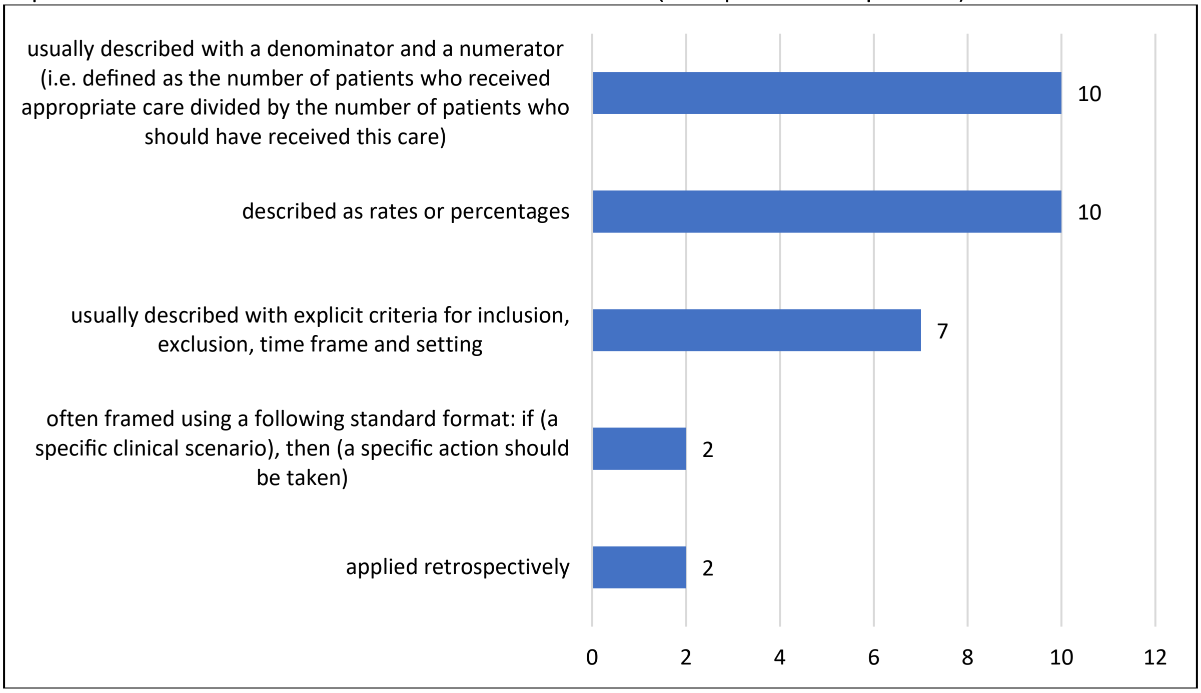

Aspect 12. Quality dimensions: Should the definition of the tool(s) include quality dimensions defined by IOM/ WHO?
(i.e. patient safety, effectiveness, equity, patient-centeredness, timeliness and efficiency)

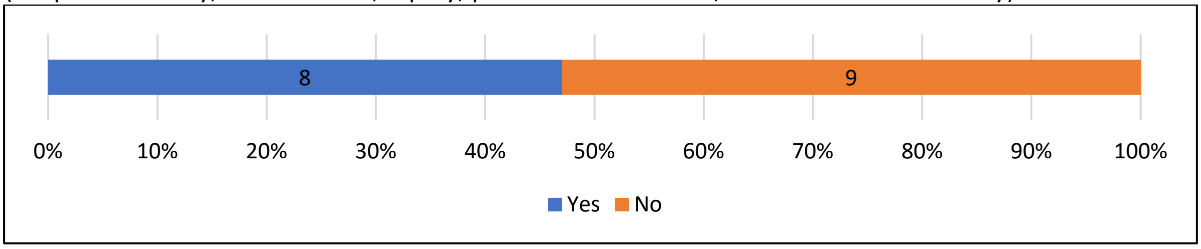

Aspect 13. Users: Who are the users of QI scores? (multiple answers possible)

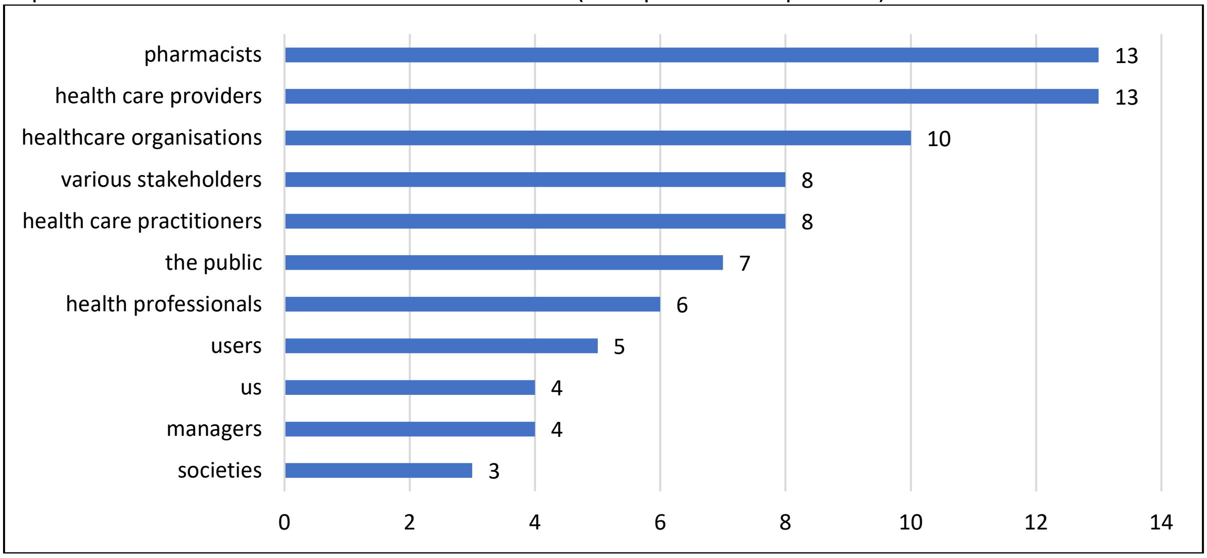

Aspect 14. Unit of analysis: What is the unit of analysis? (multiple answers possible)

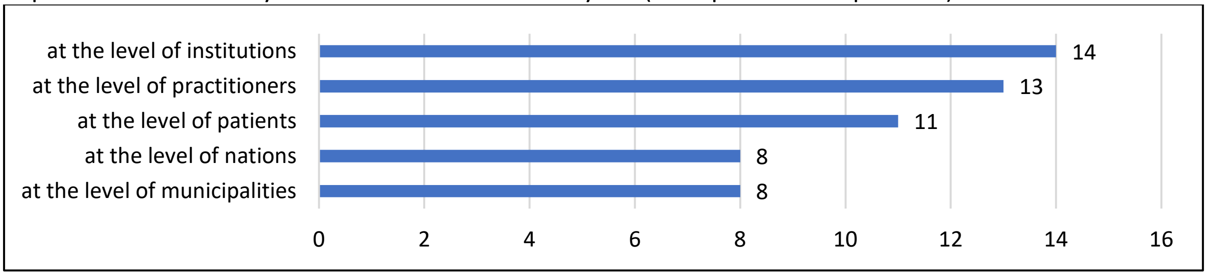

Aspect 15. Data sources: What data sources are needed? (multiple answers possible)

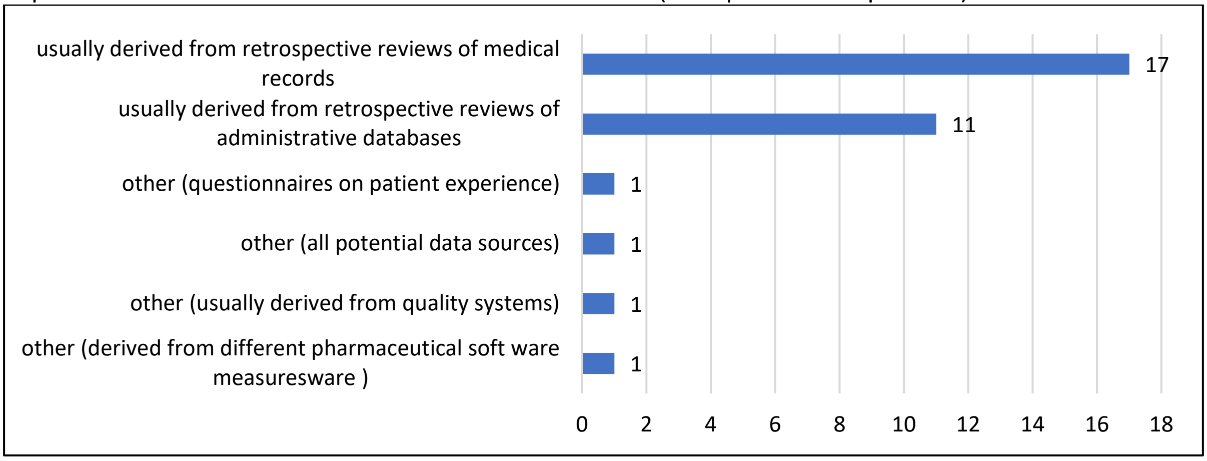

Aspect 16. Development methods: How are QIs developed? (multiple answers possible)

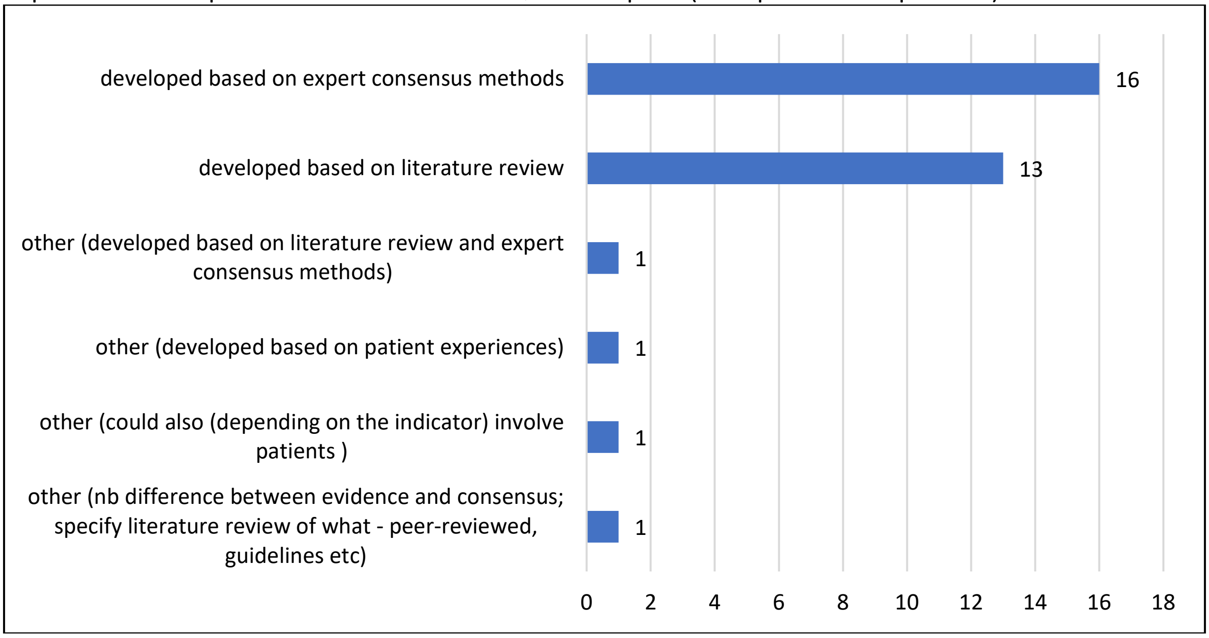

Supplement: Supplementary file 2 — Supplementary file2 (PDF 165 kb) [file 11096_2023_1631_MOESM2_ESM.pdf]
