# Supplementary material for: Pharmaceutical Care Network Europe definition of quality indicators for pharmaceutical care: a systematic literature review and international consensus development
Source: Int J Clin Pharm. 2023 Aug 30;46(1):70–9. doi: 10.1007/s11096-023-01631-8 (PMC10830737; doi:10.1007/s11096-023-01631-8)

Supplementary material C. 2nd round results

(1st version) PCNE definition of quality indicators for pharmaceutical care

Quality indicators to monitor and improve pharmaceutical care are consensus-based measures of structures and processes in the pharmacies and of outcomes in patient populations, usually described by a denominator and a numerator

Discussion point 1:
Is it necessary to include the "characteristics" aspect: 'usually described by a denominator and a numerator' -as this does not apply to the measurement of structure indicators?

Discussion summary:
In relation to the characteristic aspects "usually described by a denominator and the numerator", it's a good idea to keep a definition as simple as possible without losing details. So the characteristics here, they are important but not actually needed in the definition.
Therefore, we should delete this.

Do you agree with this comment?
"usually described by a denominator and the numerator" should be deleted.

Median	Disagreement	Result
9	No	Agreement

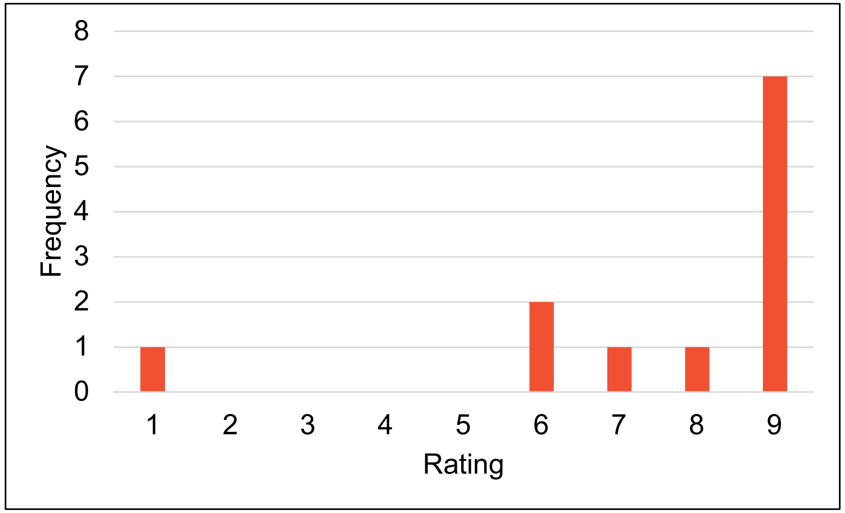

Agreement: panel median of 7-9, without disagreement
Disagreement: number of panelists rating in each extreme (1-3 and 7-9) ≥ 4

(1st version) PCNE definition of quality indicators for pharmaceutical care

Quality indicators to monitor and improve pharmaceutical care are consensus-based measures of structures and processes in the pharmacies and of outcomes in patient populations, usually described by a denominator and a numerator

Discussion point 2:
In terms of the 'development method', should we include not only "consensus-based" but also "based on literature review" in the definition?

Discussion summary:
"Consensus-based" measures is just one way to arrive at the QI. The notion of having exclusively "consensus-based" measure in the definition might not be appropriate in every case. Given evidence could be created by expert consensus and literature review, "evidence-based" is probably a better term than "consensus-based". However, strong level of evidence is not always available for QIs, especially for structure indicators. In addition, evidence can change all the time. 'Validated" measures is probably a better term than 'evidence-based' measures. 'Evidence-based' is connected to a guideline or a therapeutic option while "validated' is the term which links to a measurement tool, like a survey, measurement of adherence, or a measure of quality of life. You can have a face-validated structure indicator, which is a very low level of validation and all the way through to QIs with high predictive validity. Therefore, "consensus-based" should be replaced with "validated".

Do you agree with this comment?
"consensus-based" should be replaced with "validated".

Median	Disagreement	Result
9	No	Agreement

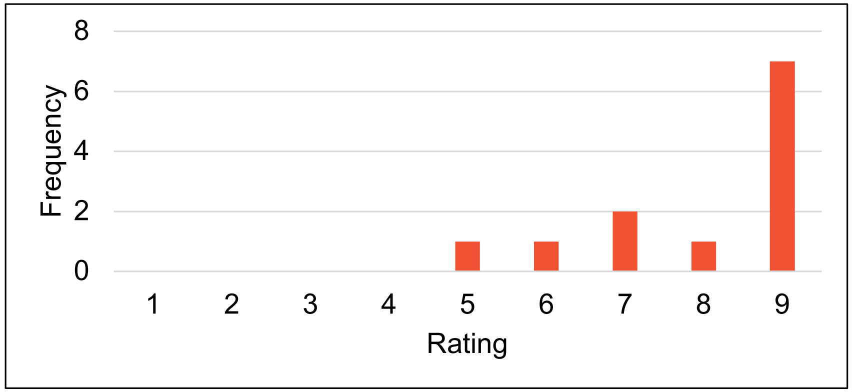

(1st version) PCNE definition of quality indicators for pharmaceutical care

Quality indicators to monitor and improve pharmaceutical care are consensus-based measures of structures and processes in the pharmacies and of outcomes in patient populations, usually described by a denominator and a numerator

Discussion point 3:
Who is the target population? (e.g. patients, individuals, persons)

Discussion summary:
Pharmaceutical care is not just provided to people who were sick. Care could be for maintaining wellness, screening or prevention. In addition, a patient with high blood pressure doesn't consider themselves a patient. All of the time they consider themselves an individual even when they're sick. Given the PCNE definition of Pharmaceutical Care has an "individuals" rather than "patients" nor "persons", "individuals" is a better word.

Therefore, the target population should be "individuals" rather than "patient populations".

Do you agree with this comment?
The target population should be "individuals" rather than "patient populations

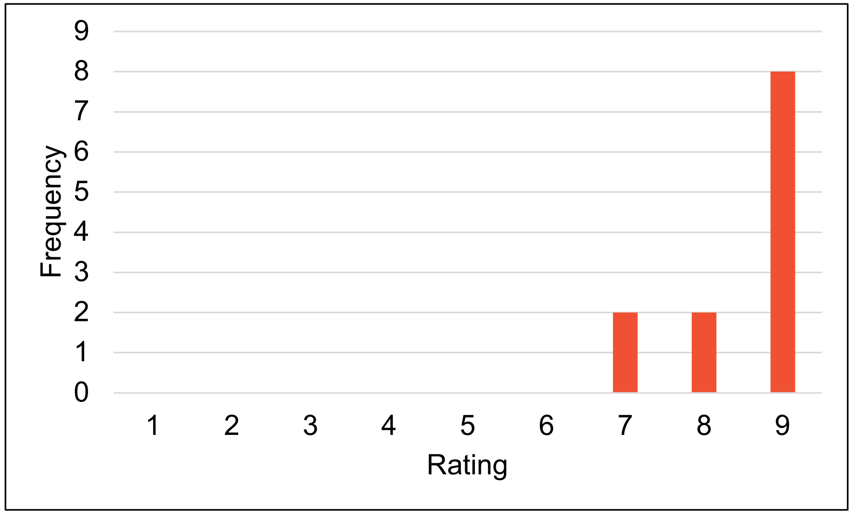

Median	Disagreement	Result
9	No	Agreement

(1st version) PCNE definition of quality indicators for pharmaceutical care

Quality indicators to monitor and improve pharmaceutical care are consensus-based measures of structures and processes in the pharmacies and of outcomes in patient populations, usually described by a denominator and a numerator

Discussion point 4:
Are the indicators actually measures or measurement tools?

Discussion summary:
"Measures" is the data you get through using quality indicators. QIs are probably "measurement tools" rather than "measures". Therefore, the term "measures" should be replaced with "measurement tools".

Do you agree with this comment?
The term "measures" should be replaced with "measurement tools".

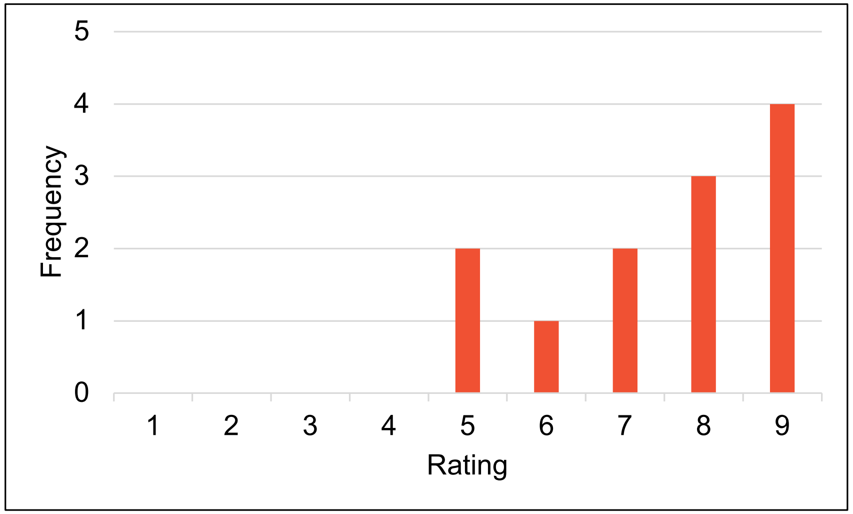

Median	Disagreement	Result
8	No	Agreement

(1st version) PCNE definition of quality indicators for pharmaceutical care

Quality indicators to monitor and improve pharmaceutical care are consensus-based measures of structures and processes in the pharmacies and of outcomes in patient populations, usually described by a denominator and a numerator

Discussion point 5 & 6:
Pharmaceutical care may be delivered not in the pharmacy but in the clinic or outside of the room or at discharge in a hospital. It's not restricted to in the pharmacies.
Discussion summary:
Because quality indicators could be for GPs and other healthcare professionals, the definition should start with "quality indicators for pharmaceutical care". If we do that, we can skip the term 'in the pharmacies' instead of specifying where the structures and processes are provided because the definition implies that structures and processes "in the delivery of pharmaceutical care".
Because we have already defined pharmaceutical care, we don't need to include what pharmaceutical care is in the definition of quality indicators.

Do you agree with these comments?
The definition should start with "Quality indicators for pharmaceutical care".

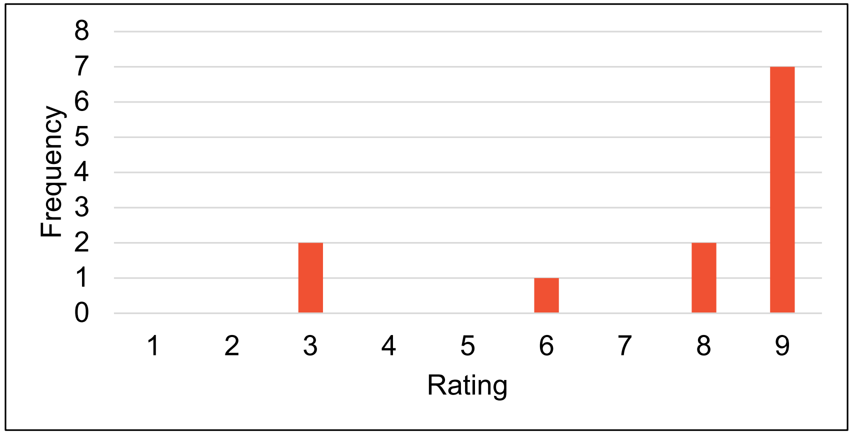

Median	Disagreement	Result
9	No	Agreement

(1st version) PCNE definition of quality indicators for pharmaceutical care

Quality indicators to monitor and improve pharmaceutical care are consensus-based measures of structures and processes in the pharmacies and of outcomes in patient populations, usually described by a denominator and a numerator

Discussion point 5 & 6 (*continued*):

Do you agree with these comments?
We should delete the terms "in the pharmacies" instead of specifying where the structures and processes are measured.

Median	Disagreement	Result
9	No	Agreement

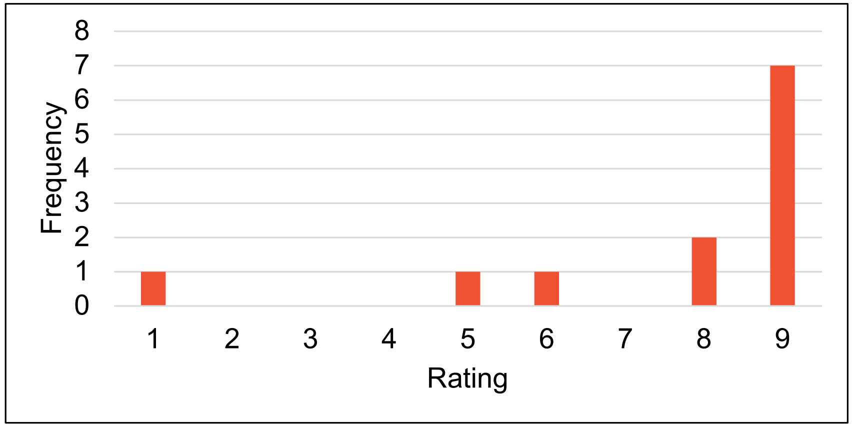

(1st version) PCNE definition of quality indicators for pharmaceutical care

Quality indicators to monitor and improve pharmaceutical care are consensus-based measures of structures and processes in the pharmacies and of outcomes in patient populations, usually described by a denominator and a numerator

Discussion point7:
Are the purposes of using QIs to “monitor and improve” quality of care?
Discussion summary:
The idea behind quality indicators is of course to improve. However, quality can also decrease. So in that way, we are “monitoring” quality of care using indicators. Therefore, we should remove the term “improve” from the definition.

Do you agree with this comment?
We should remove the term "improve" from the definition

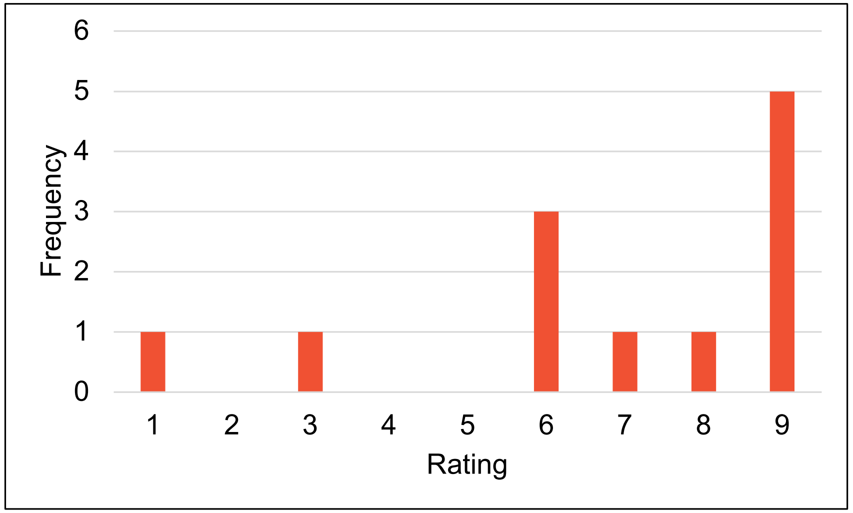

Median	Disagreement	Result
7.5	No	Agreement

(2nd version) PCNE definition of quality indicators for pharmaceutical care

Quality indicators for pharmaceutical care are validated measurement tools to monitor structures, processes or outcomes in the context of care provided by pharmacists

Updated definition (8/8)

Considering the discussion points described so far, we updated the definition of quality indicators for pharmaceutical care as following:

Do you agree with the definition of quality indicators for pharmaceutical care as stated above?

Median	Disagreement	Result
7	No	Agreement

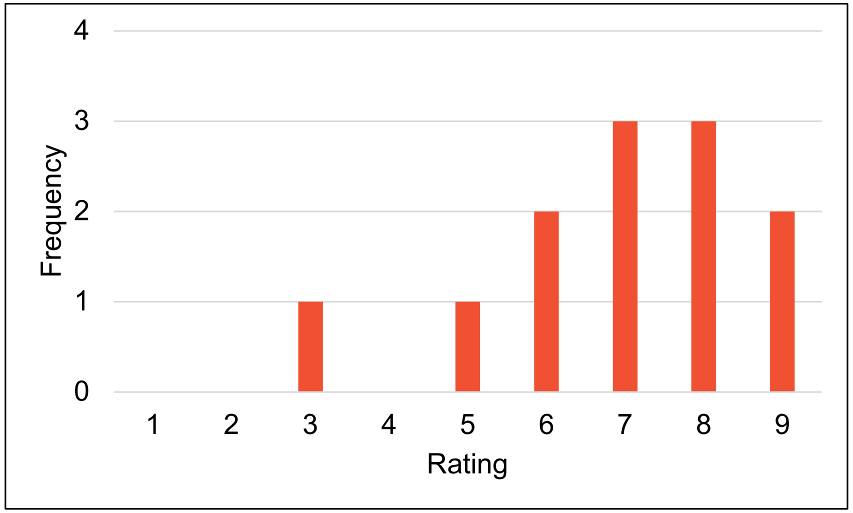

Supplement: Supplementary file 3 — Supplementary file3 (PDF 930 kb) [file 11096_2023_1631_MOESM3_ESM.pdf]
